# Measurement of Early Disease Blueberries Based on Vis/NIR Hyperspectral Imaging System

**DOI:** 10.3390/s20205783

**Published:** 2020-10-13

**Authors:** Yuping Huang, Dezhen Wang, Ying Liu, Haiyan Zhou, Ye Sun

**Affiliations:** 1 College of Mechanical and Electronic Engineering, Nanjing Forestry University, Nanjing 210037, China; huangyuping@njfu.edu.cn (Y.H.); dezhenwang0@gmail.com (D.W.); liuying@njfu.edu.cn (Y.L.); zhouhaiyanzj@njfu.edu.cn (H.Z.); 2 College of Engineering, Nanjing Agricultural University, Nanjing 210031, China

**Keywords:** early disease blueberry, hyperspectral imaging, identification, effective wavelength selection

## Abstract

Blueberries, which are rich in nutrition, are susceptible to fungal infection during postharvest or storage. However, early detection of diseases in blueberry is challenging because of their opaque appearance and the inconspicuousness of spots in the early stage of disease. The goal of this study was to investigate the potential of hyperspectral imaging over the spectral range of 400–1000 nm to discriminate early disease in blueberries. Scanning electron microscope observation verified that fungal damage to the cellular structure takes place during the early stages. A total of 400 hyperspectral images, 200 samples each of healthy and early disease groups, were collected to obtain mean spectra of each blueberry samples. Spectral correlation analysis was performed to select an effective spectral range. Partial least square discrimination analysis (PLSDA) models were developed using two types of spectral range (i.e., full wavelength range of 400–1000 nm and effective spectral range of 685–1000 nm). The results showed that the effective spectral range made it possible to provide better classification results due to the elimination of the influence of irrelevant variables. Moreover, the effective spectral range combined with an autoscale preprocessing method was able to obtain optimal classification accuracies, with recognition rates of 100% and 99% for healthy and early disease blueberries. This study demonstrated that it is feasible to use hyperspectral imaging to measure early disease blueberries.

## 1. Introduction

Blueberries are popular among consumers because of their appealing flavor and high nutraceutical value, including anthocyanin, flavonol, and vitamin [1,2]. Most fresh-market blueberries are harvested during hot and rainy summers, and are hand-picked. However, blueberries have thin skin and soft flesh, which makes it easy for them to be infected by pathogenic microorganisms, and they are prone to bruising damage during picking, packaging and transportation [3], which also facilitates the invasion of pathogens. Infected blueberries can neither be stored for a long time nor transported long distances, and one decayed blueberry can infect a whole consignment [4]. Therefore, one of the main tasks in post-harvest processing in the blueberry industry is to detect the decayed ones as early as possible.

As the skin of the blueberry is purple-black and opaque to most visible light, the defect region below the skin is not visible to the human eye in the early stage of disease [5]. Therefore, it is a challenging task to use traditional RGB images to accurately detect disease. Several nondestructive techniques, including thermographic imaging, low-field nuclear magnetic resonance, computer vision and multi- and hyper-spectral imaging, have been investigated for quality detection of blueberries, such as: bruising [6,7], maturity stages [8], mechanical damage [9], soluble solids content [10,11] and firmness [12]. Numerous studies have been carried out for blueberry defects originating from bruising damage; however, few studies have reported on the detection of disease. Therefore, it is very important to find a suitable technique to achieve rapid and effective blueberry disease detection.

Visible and near-infrared spectroscopy is an excellent tool for assessing quality and safety of fruit, since the obtained light contains information on the interaction of light with the tissue of the samples over the wide spectral range [13,14,15,16]. The technique is advantageous due to its fast and little sample preparation, and it is also applied in the evaluation of blueberry quality [17,18,19]. However, the visible and near-infrared technique measures light reemitted from a specific area of the sample without offering spatial information, which has limitations for the accurate detection of blueberries.

Hyperspectral imaging, which combines imaging and spectral information to detect external or internal quality attributes, is a promising detection method [20,21,22]. Furthermore, spectral signatures can be used to uniquely characterize, identify and quantify the chemical composition of agricultural products [23,24,25,26], which has advantages in the detection of some early defects that are invisible to the naked eye. In the last decade, numerous studies have been reported on the detection of diseases in fruit using hyperspectral imaging. A study by Pan et al. [27] investigated the application of hyperspectral imaging for monitoring the pathogenetic process and early detection of disease in pears, and the results reported that they achieved an overall accuracy of 97.5% for detection of early pear disease. Pieczywek et al. [28] combined hyperspectral imaging and chlorophyll fluorescence for the early detection of fungal infections in stored apple. The results showed that hyperspectral imaging enabled the statistically significant detection of infected areas as early as 2 days after inoculation, which is invisible to the naked eye. Sun et al. [4] discriminated three kinds of diseases in peaches using hyperspectral imaging techniques, and studied the detection effect of hyperspectral technology on different degrees of diseases. Li et al. [29] demonstrated the usability of hyperspectral imaging for automatic detection of early symptoms of decay caused by the *Penicillium digitatum* fungus in citrus fruit. Despite the progress in applying hyperspectral imaging for disease detection in other agricultural products, there are still many challenges that must be addressed for its application to blueberries. Furthermore, due to the amounts of multidimensional data, data preprocessing, feature extraction and optimal wavelength selection are very important components in hyperspectral data analysis. Prior studies have shown that suitable preprocessing methods on data lead to better results for further prediction/classification [30,31,32,33], and band selection eliminates low signal-to-noise ratio or low sensitivity wavebands, which will increase the inversion accuracy and decrease the computation time. Noviyanto and Abdulla [34] investigated hyperspectral imaging in building a botanical origin classification model for honey based on reflectance spectra, and the proposed data processing methods showed promising results, including noisy band elimination, spectral normalization and hierarchical classification. Fu et al. [35] proposed a diversity of strategies to progress of hyperspectral data processing and modelling for cereal crop nitrogen monitoring, and analyzed in detail the determination of optimal wavelet, scale and wavelength in continuous wavelet transformations.

So far, hyperspectral imaging has been explored for detection of bruising, along with several quality parameters of blueberries. The objective of this study was therefore to demonstrate the capability of hyperspectral imaging for the detection of disease-infected blueberries at early stages. The specific objectives of the research were to: (1) measure the hyperspectral imaging data of healthy and early disease blueberries; (2) observe the microstructural changes of early disease blueberry; (3) select the optimal preprocessing methods and effective spectral range for disease detection of blueberries; and (4) classify the early disease fruit based on the spectral information.

## 2. Materials and Methods

### 2.1. Blueberry Samples and Evaluation of Early Disease

**Figure 1:**
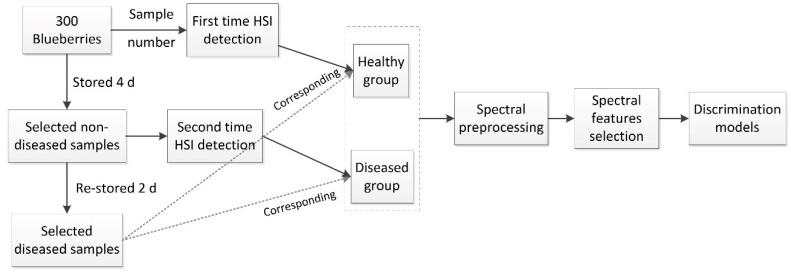
Flowchart of analytical procedure for disease detection.

‘Wanqi’ blueberries purchased from a local fruit shop were used in this research. Three hundred blueberries with no visible defects and/or contamination were selected, and all samples were numbered one by one. Then, the healthy blueberries were scanned using the hyperspectral imaging system. After being scanned for the first time, all blueberries were stored at 20 °C in an incubator at 85–90% relative humidity to obtain natural disease samples. After the fourth day of storage, a portion of the samples were naturally decayed, the blueberries that were badly rotten were removed, and the remaining samples were scanned for the second time using hyperspectral imaging. After being scanned for the second time, the remaining blueberries were stored in the incubator again. After a further two days of storage, they were taken out to observe whether disease had occurred; the samples without decay were removed, and the labels of the remaining diseased samples were recorded as usable samples. Finally, a total of 200 blueberries were selected; that is, the first set of hyperspectral data, corresponding to the 200 samples, comprised the healthy group, while the second set of hyperspectral data comprised the early disease group. A thorough description of the sample processing is provided as a flowchart in Figure 1.

### 2.2. Hyperspectral Imaging Acquisition

**Figure 2:**
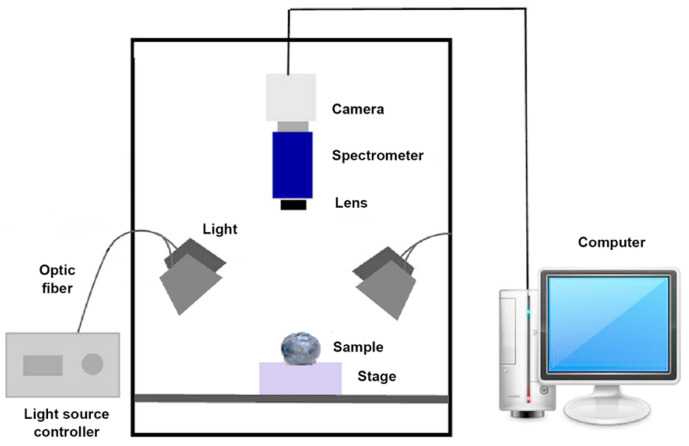
Schematic of the hyperspectral imaging system.

Hyperspectral images were obtained for each of the blueberries using a hyperspectral imaging system in reflectance mode, which consisted of an imaging spectrograph (ImSpector V10E, Specim, Finland), a camera (Andor Zyla, Oxford, Mumbai) covering a spectral region of 400–1000 nm with a spectral resolution of 2.8 nm, four adjustable regulated halogen tungsten lamps with 100 W, a horizontal motorized stage (HSIA-T1000-IMS, GaiaSorter, Chengdu, China) and a focal length lens, as shown in Figure 2. The distance between the blueberry sample and the camera was set at 5 cm, the hyperspectral imaging system was set with an exposure time of 20 ms, and adjusted conveyor speed was set at 0.36 cm/s.

A total of 400 hyperspectral images, 200 each of the two grades (i.e., healthy and early disease), were used in data analysis. The reflectance calibration should be performed by acquiring dark and white hyperspectral images to account for the background spectral response of the instrument and the dark current of the camera. The dark reference image was obtained by completely covering the camera lens with an opaque cap, and a white reference image was acquired using a Teflon white board (HSIA-LA-TS-30, GaiaSorter, Chengdu, China). These two images were then used to calculate the pixel-based relative reflectance for the sample images using the following formula:

(1) $$R=\frac{R_{0}-R_{d}}{R_{w}-R_{d}}$$

where *R* is the relative reflectance image for sample, *R*_0_ is the raw reflectance image, *R_d_* is the dark reference image, and *R_w_* is the white reference image. The relative images were used for further analysis. The entire area of the blueberry in the corrected images was selected as the region of interest (ROI) using ENVI software (ENVI4.7, Research System Inc., Boulder, CO, USA). In total, 400 mean reflectance spectra were obtained from the ROIs of the samples for data analysis.

### 2.3. Microstructural Analysis

Right after the hyperspectral imaging, three blueberries were randomly selected from the two groups for microstructural analysis. Scanning electron microscopy (SEM) (Quanta 200, Thermo Fisher Scientific, Hillsboro, OR, USA) were conducted on the tissue specimens to quantify the microstructural changes of peel and pulp for pathogen infection. Healthy and diseased tissue specimens, each being 1 cm in length, 1 cm in width and 1 mm in thickness, were prepared from the peel and pulp of the healthy and diseased areas, respectively, and they were then quickly immersed and fixed in 2.5% glutaraldehyde solution at 4 °C. The fixed specimens were then washed with 0.1 M phosphate buffer, and post-fixed in 1% phosphate buffered osmium tetroxide (OsO4) solution for 1 h at 0 °C. After another wash with phosphate buffer, they were dehydrated with a series of ethanol concentrations. The fixed and dehydrated specimens were quench-cooled in nitrogen slush under vacuum and coated with gold/palladium.

### 2.4. Data Analysis and Chemometrics

The 400 blueberry samples were randomly divided into two sets, with 50% (200 samples) for training and the remaining 50% (200 samples) for independent testing. Spectral preprocessing was performed to eliminate potential variability among samples due to scattering and optical interference, as well as to improve the accuracy and reliability of the models [36]. In this study, ‘no spectral preprocessing’, ‘autoscale’ and ‘log(1/R)’ were analyzed and compared. Autoscale is commonly used preprocessing method for scaling each variable to unit standard deviation. It is a valid approach for correcting different variable scales and units if the predominant source of variance in each variable is signal rather than noise. The early disease blueberries should have different constituents in the tissue to the healthy ones, and absorbance log(1/R) is more sensitive to chemical composition, which means log(1/R) is a possible valid method for improving model performance. Partial least squares discriminant analysis (PLSDA) is a multivariate inverse least squares discrimination method, which calculates the probability of each sample belonging to each possible class. PLSDA is capable of extraction of spectral features and removing interference from partial noises; however, a PLSDA model established by full wavelength may end up being computationally intensive and complex, and if there is a low signal-to-noise ratio, or spectral overlaps appear in the full wavelength, it will be too difficult for the model to reach optimal performance. Therefore, it is necessary to remove irrelevant or non-linear variables, and extract effective spectral information to make models more robust and simplified. PLSDA models were developed using MATLAB R2017a (The MathWorks, Inc., Natick, MA, USA), coupled with PLS Toolbox 8.6 (Eigenvector Research, Inc., Wenatchee, WA, USA). Venetian blinds cross-validation is simple and easy to implement, and is generally safe to use if there are relatively large samples that are already in random order. It was used to determine the optimal number of latent variables based on the minimum classification error of cross validation. The performance of the models was evaluated by classification accuracy (recognition rate) for calibration, cross-validation and test sets of samples.

## 3. Results and Discussion

### 3.1. Disease Evaluation and SEM Observation

After the fourth day of storage, twenty-seven blueberries that were badly rotten were removed, and for the remaining samples without obvious hyphae, it was difficult to distinguish whether they were infected. Then, after re-storing for two days, two hundred blueberries that had become rotten were selected and identified as the early disease samples that had been infected on the fourth day, and seventy-three blueberries with no disease were eliminated. Therefore, these two hundred blueberries were used as the healthy group at the first day and early disease group at the fourth day.

**Figure 3:**
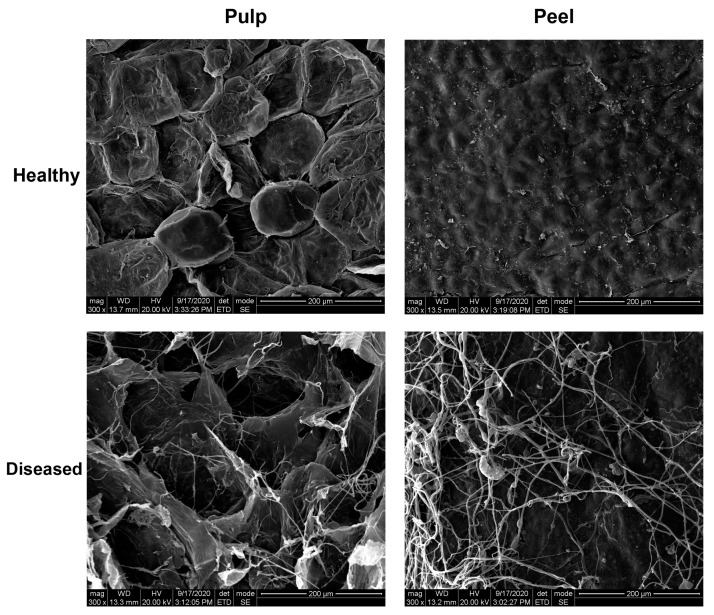
Scanning electron microscopy images of surface cellular characteristics for the pulp and peel tissues of healthy and diseased blueberry.

It is easy for fungi to invade tiny areas of mechanical damage (for example: damage to the carpopodium caused by picking), and their growth normally occurs in those areas of fruit [37]; it is often possible to observe the fungal development directly with the naked eye. However, their presence is invisible to the unaided eye during the early stages of development, while the damage they cause to the cell microstructure is still significant [38]. SEM observations were performed on the peel and pulp of healthy blueberries (i.e., on the first day) and slightly decayed blueberries (i.e., on the fourth day of natural decay), respectively (Figure 3). In the healthy sample, no hyphae were observed inside or outside the peel, the cell structure were normal, and the cell membrane was integrated. Although no fungal infection symptoms could be observed on the surface of blueberries with the naked eye at the early infection stage, unknown fungal hyphae were observed using SEM. It can been seen that an interconnected network of hyphae (mycelium) covered over the cell structure for the pulp and peel, and destroyed most of the cell membrane. Therefore, although some blueberries looked healthy, the internal microstructure had actually been seriously damaged. With scanning electron micrographs, it was possible to define the occurrence of spore germination and hyphae growth in correspondence to the early disease of the samples in which no growth could be recorded by the naked eye. The light based on hyperspectral imaging technique can reflect changes in internal structure, so it can effectively reflect diseased samples.

### 3.2. Spectral Correlation Analysis and Effective Spectral Range Selection

**Figure 4:**
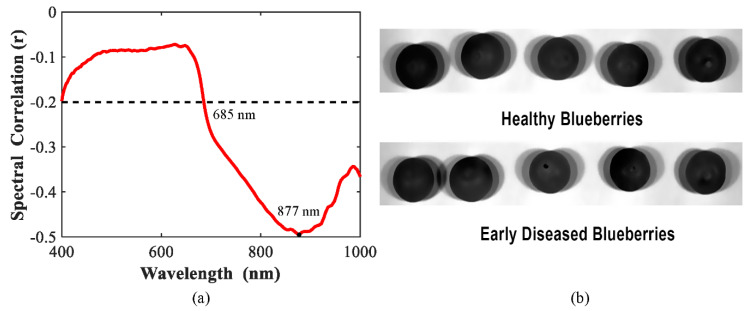
Correlation curves for blueberry between grading class and relative reflectance of individual wavelengths for the visible and shortwave near-infrared region of 400–1000 nm (**a**), and the hyperspectral images for healthy and early disease blueberries in 877 nm (**b**).

The correlation curve between the blueberry grades (0: healthy, 1: early disease) and the single wavelengths of the visible and shortwave near-infrared (Vis/SWNIR) spectra is presented in Figure 4a. It is easy to observe that there were negative correlations between the blueberry grades and the single wavelengths over the full spectral range of 400–1000 nm, indicating that as the reflectance increased, the grades turned from 1 to 0, meaning that the reflectance for healthy samples was higher than for early disease samples. This is also demonstrated in the next section, shown in Figure 4a. Moreover, the correlations varied with wavelength bands, with some wavelength bands having higher correlations, such as the spectral region of 685–1000 nm, which suggested the possibility of better classification for blueberries using a few selected wavelengths. Relatively higher correlations appeared at 750–1000 nm, while in the visible region, the correlations were lower. On the one hand, the obtained reflectance for blueberry in the visible region had lower signal-to-noise ratio, as shown in Figure 4a. On the other hand, the light in the visible region is better for assessing pigments in the tissue due to the fact that the vibrations of the wavelength in the visible region give humans a sensation of color, while NIR light has a strong interaction with hydrogen bonds, like N-H, C-H, O-H, etc., in fruit products, yielding different spectral patterns according to changes in the structural and chemical properties. It is possible that there are no distinct color differences between healthy and early disease blueberries; however, the chemical compositions and structural properties should be different between these two grades, which could partly explain the higher correlations that appeared in the NIR region. Figure 4b displays the hyperspectral images of the healthy and early disease blueberries at the wavelength of 877 nm, with highest spectral correlation. There were no prominent differences (i.e., color differences or external defective differences) between these two images, suggesting that it could be difficult to identify early disease samples based on the information in the image.

As discussed above, in order to improve model performance, extracting effective spectral information and removing irrelevant variables is necessary in order to simplify the models and enhance the classification results of models. Spectral correlations lower than 0.2 were removed for wavelength selection, which means the remaining spectral region of 685–1000 nm was used for model building.

### 3.3. Spectral Features Related to Early Disease

**Figure 5:**
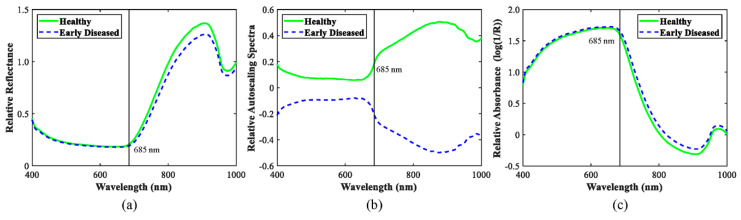
The mean relative reflectance (**a**), autoscaling (**b**), and absorbance spectra (**c**) for healthy and early disease blueberries.

Figure 5 illustrates the mean relative spectra for healthy and early disease blueberries of three spectral preprocessing methods over the spectral range of 400–1000 nm. There were two noticeable absorption peaks for the mean spectra of both healthy and early disease blueberries at around 680 nm, due to chlorophyll, and 970 nm, due to water or O-H functional groups [24,25]. There were no significant differences in the spectral range of 400–685 nm between healthy and early disease fruit, which could be due to the dark color of blueberries, resulting in difficult-to-observe pigment changes. However, distinct differences were observed between healthy and early disease blueberries for the spectra beyond 685 nm. The interaction of light with the blueberry sample involves absorption and scattering, Lu [39] reported that absorption is dependent on the chemical constituents, while scattering is influenced by the cell structure and extra- and intra-cellular matrices of fruit tissues. Light absorption was more prominent in early disease blueberries in the NIR region, which could be due to the changes in chemical composition (like chlorophyll, sugar and other nutrients degradation) and structure (cell membrane damage, as shown in Figure 3). In addition, differences in the mean spectra between healthy and early disease blueberries were also observed for the three spectral preprocessing methods; autoscale preprocessing showed the largest distinctions in the mean spectra between healthy and early disease blueberries, which suggests that this spectral preprocessing method could be more appropriate for early disease blueberry detection. Moreover, the spectral patterns between relative reflectance without preprocessing and absorbance were totally opposite.

### 3.4. Discrimination Models for Early Disease Blueberry Detection

Table 1 summarizes the total classification results for the training and test sets. The spectra without preprocessing overall had lower discrimination accuracies, which indicated that appropriate preprocessing methods could further improve classification results. In the full wavelength range, the autoscale preprocessing method showed 3.7%, 4.4% and 4% improvement on the calibration, cross-validation (CV) and test sets, while log(1/R) showed 2.7%, 5.5% and 4.6% improvement on these three sets. Removing the irrelevant variables is one of the most effective methods for enhancing the performance of discrimination models, and could simplify the calculation of models and improve the mathematical model’s robustness and reliability. In this study, after eliminating the variables over spectral range of 400–685 nm, all of the accuracies for spectra with or without preprocessing methods were improved, especially for the spectra with the no preprocessing method, with improvements of 5.3%, 6.6% and 12% for calibration, CV and test sets compared with spectra over the full wavelength range. Unlike in the full spectral region, after doing wavelength selection, the spectra with log(1/R) had similar accuracies those with the no preprocessing method. The selected spectra (685–1000 nm) with the autoscale method provided the best classification results, with total recognition results of 0.995 in the test set, suggesting that the autoscale method could be a useful means for enlarging the differences in the blueberry samples.

Further breakdowns of the classification results for healthy and early disease blueberries by three preprocessing methods based on PLSDA models are presented in Table 2. In the full wavelength range of 400–1000 nm, classification accuracies for healthy blueberries were consistently higher than those for early disease blueberries, for both training and test sets, which could be attributed to the relatively homogenous tissues of the healthy blueberries. In the training set, the classification accuracies for recognition of healthy and early disease blueberries were higher than 0.90; however, the recognition rates for early disease blueberries in the test set were lower than 0.90, which indicated that the built models did not have enough adaptability. As discussed above, the spectral preprocessing methods were able to enhance the classification accuracies. In the training set, the spectral preprocessing methods presented improvements between 2.0% and 6.4% for the healthy samples, and 4.6% and 5.7% for the early disease samples. In the test set, the improvements for healthy blueberries were better than those of the training set; however, the best improvement for the early disease samples was only 2.2%.

Wavelength selection could further enhance the performance of PLSDA models. The accuracies for the early disease blueberries were similar, or even better than those of the healthy samples, which suggested the the visible spectral range of 400–685 nm was not suitable for discrimination of the diseased blueberries. Additionally, the recognition rates in the test set for the early disease blueberries increased significantly, with improvements of 15.7%, 14.5% and 12.1% for the no spectral preprocessing, autoscale and log(1/R) methods. Moreover, the relatively high classification results for both healthy and early disease blueberries, with over 0.98 accuracy in the calibration set and over 0.95 accuracy in the CV and test sets, suggested that the established PLSDA models were stable and robust. Log(1/R) had similar accuracies in the test set to the no spectral preprocessing method, but log(1/R) had the best classification results in the cross-validation set, since a total of only three samples were misclassified: two healthy samples were alotted to the early disease group, and one early disease sample was alotted to the healthy group. Furthermore, the accuracy in the calibration set also presented better results, which demonstrated that the spectral preprocessing methods were capable of improving the performance of the PLSDA models. Overall, the autoscale methods showed the best classification results for identifying the healthy and early disease blueberries accurately, with 100% recognition rates in the calibration set, and with recognition rates for healthy and early disease blueberries of 100% and 99% in the test set.

The classification results for the recognition of early disease blueberries were also comparable or even better that reported in previous studies. Qiao et al. [40] classified early decayed blueberries using hyperspectral images coupled with improved deep residual 3D convolutional neural network algorithms, and reported maximum classification results of 95.42%. Jiang et al. [41] proposed nondestructive detection of blueberry bruising using a NIR hyperspectral reflectance imaging support vector machine (SVM) model, and obtained a maximum classification accuracy of 96%. Fan et al. [42] applied NIR hyperspectral reflectance imaging with optimum wavelengths to detect internal bruising for blueberries, with overall discrimination accuracies for healthy and bruised blueberries in the validation set of 93.3% and 98.0%.

## 4. Conclusions

In this paper, a hyperspectral imaging system over the spectral range of 400–1000 nm was applied for identification of early disease blueberries. The mean spectra for each of the blueberry samples were extracted from the hyperspectral images, and the effective spectral range was selected based on spectral correlations. The spectral correlation curves indicated that higher correlations were shown in the range of 685–1000 nm; additionally, the established PLSDA models also gave better classification accuracies for recognition of early disease blueberries. Spectral preprocessing methods were also employed to analyze and compare the classification results. The results demonstrated that the autoscale spectral preprocessing method combined with effective wavelength (685–1000 nm) presented optimal classification, with recognition rates of 100% and 99% for healthy and early disease blueberries, respectively. In addition, the scanning electron microscopy images for healthy and early disease blueberries were obtained to observe the differences in the view of microstate. Early disease blueberries did not appear to have obvious spoilage mold, but a lot of hyphae were found on the peel and pulp on the SEM images, resulting in different chemical compositions and structural characteristics in the tissue, which could also explain the good classification results for the early disease blueberries obtained by hyperspectral imaging technique. This study demonstrated that hyperspectral imaging in the spectral range of 400–1000 nm has potential for use in the detection of early disease blueberries.

## Figures and Tables

**Table 1:** Total classification results for recognition of blueberry based on partial least squares discriminant analysis models over the spectral range of 400–1000 nm and 685–1000 nm.

Spectral Type	Spectral Preprocessing	Total Accuracy		
		Training Set		Test Set
		Calibration	Cross-Validation	
Full (400–1000 nm)	None	0.935	0.910	0.875
	Autoscale	0.970	0.950	0.910
	Log(1/R)	0.960	0.960	0.915
Selected (685–1000 nm)	None	0.985	0.970	0.980
	Autoscale	1.000	0.975	0.995
	Log(1/R)	0.995	0.985	0.980

**Table 2:** Classification results for healthy and early disease blueberries by using partial least squares discriminant analysis over the spectral range of 400–1000 nm and 685–1000 nm *.

Spectral Type	Spectral Preprocessing	Training Set						Test Set		
		Calibration			Cross-Validation					
		H	D	Accuracy	H	D	Accuracy	H	D	Accuracy
Full	None	99	8	0.952	95	9	0.913	86	15	0.896
		5	88	0.917	9	87	0.906	10	89	0.856
	Autoscale	101	3	0.971	99	5	0.952	92	14	0.958
		3	93	0.969	5	91	0.948	4	90	0.865
	Log(1/R)	101	5	0.971	101	5	0.971	92	13	0.958
		3	91	0.948	3	91	0.948	4	91	0.875
Selected	None	102	1	0.981	100	2	0.962	93	1	0.969
		2	95	0.990	4	94	0.979	3	103	0.990
	Autoscale	104	0	1.000	101	2	0.971	96	1	1.000
		0	96	1.000	3	94	0.979	0	103	0.990
	Log(1/R)	103	0	0.990	102	1	0.981	94	2	0.979
		1	96	1.000	2	95	0.990	2	102	0.981

* H: healthy; D: early disease.
